# Urinary Diversion Can Improve the Chance of Implementing New Therapeutic Lines in Patients with Malignant Ureteral Obstruction: A Multicenter Study

**DOI:** 10.3390/curroncol31110523

**Published:** 2024-11-13

**Authors:** Marcelo Cartapatti, Roberto Dias Machado, José Carlos Mesquita, Raphael Freua, Diego Cáceres, Rodolfo Borges dos Reis

**Affiliations:** 1 Clinics Hospital, Ribeirão Preto Medical School, University of São Paulo, Ribeirão Preto 05508-220, SP, Brazil; rodolforeis@fmrp.usp.br; 2 São José do Rio Preto Medical School (FAMERP), Av. Brigadeiro Faria Lima, 5416-Villa São Pedro, São José do Rio Preto 15090-000, SP, Brazil; jmesquita19@uol.com.br (J.C.M.); raphael_freua@hotmail.com (R.F.); 3 Barretos Cancer Hospital, Barretos 14784-400, SP, Brazil; robertodmachado@hotmail.com (R.D.M.); diegoderly@gmail.com (D.C.)

**Keywords:** malignancy, ureteral obstruction, urinary diversion, kidney function, survival

## Abstract

Purpose: Malignant ureteral obstruction is generally associated with a poor disease prognosis; therefore, managing these cases is challenging. We describe our experience in treating malignant ureteral obstruction with urinary diversion and the impact of these procedures on the indication for new antineoplastic therapy and survival. Materials and Methods: We retrospectively reviewed the data of patients with advanced cancer associated with malignant ureteral obstruction who underwent urinary diversion at three tertiary institutions between January 2013 and July 2022. Results: This study included 420 patients (mean age, 58.7 years (range, 18–90 years) with a mean follow-up of 20.3 months. Cervical (36.2%) and bladder cancers (18.6%) were the most prevalent primary neo-plastic sites. The mean creatinine values measured before diversion, 30 days after surgery, and most recently were 3.45, 1.84, and 2.59 mg/dL, respectively. In total, 300 patients (71.4%) received antineoplastic treatment, 195 received palliative treatment, and 105 received curative treatment. After an average of 251.87 postoperative days, 265 (64%) patients died. The mean overall survival was 610.76 days. Patients with prostate and cervical neoplasms had the most prolonged overall survival (573.13 and 549.28 days, respectively), whereas patients with bladder and colorectal cancer had the worst overall survival (480.25 and 370.53 days, respectively). Conclusions: Urinary diversion improves kidney function and opens a therapeutic window for a new line of antineoplastic therapy that provides a cure or increases patient survival.

## 1. Introduction

Upper urinary tract (UUT) obstruction is a heterogeneous clinical entity whose optimal treatment choice is often medically challenging. The degree of kidney injury, as well as the intensity and variety of associated symptoms, depends on the severity of the obstruction, the chronicity of the condition, and the basal function of the organ [1,2,3,4,5,6,7].

The actual incidence of UUT obstruction due to malignancy is still unknown, but this condition is usually associated with poor prognosis, with overall survival rates ranging from 2 to 15.3 months [8,9]. In most cases, it is a slow, progressive, and often asymptomatic process; symptoms, when present, are vague and nonspecific and can include flank discomfort, lethargy, and malaise [10,11].

Malignant UUT obstruction is a potentially life-threatening condition that is often not diagnosed promptly. Although it is frequently linked to a poor prognosis, appropriate treatment can lead to increased survival and significant improvement in the patient’s symptoms and quality of life. However, the decision regarding the actual necessity for treatment and the optimal timing involves intricate issues that encompass much more than simply diagnosing urinary obstruction.

We hypothesize that urinary diversions improve renal function in patients with malignant ureteral obstruction. This improvement enables the use of new oncological treatments, resulting in increased survival and enhanced quality of life. Our primary endpoint is to analyze the impact of urinary diversion on creating new therapy opportunities for those patients. The secondary endpoints are the overall survival and renal function improvement.

## 2. Materials and Methods

We retrospectively reviewed the data of patients with advanced tumors associated with malignant ureteral obstruction who underwent minimally invasive urinary diversion with either a ureteral stent (double J) or percutaneous nephrostomy at three public tertiary care institutions between January 2013 and July 2022. This study was approved by the ethics committee of each center. The inclusion criteria were age over 18, urinary diversion with internal or external catheter due to ureteral obstruction, oncologic condition as the cause of urinary obstruction, and presence of hydronephrosis confirmed by imaging. The exclusion criteria were as follows: patients who underwent urinary diversion after intraoperative analysis without a previous clinical evaluation, absence of complete data in the medical records, absence of hydronephrosis confirmed on imaging, and obstruction unrelated to cancer. Informed Consent Statements were not applicable, since this was a retrospective study with data regarding patients’ personal information not being collected. Due to the sample heterogeneities, we separated patients by the stage of cancer only into two groups: locally advanced and metastatic disease. We also collected data on demographics (age, sex, and comorbidities), cancer etiology, and treatment type performed.

Data on the type of drainage (double J or percutaneous nephrostomy), laterality, degree of obstruction, and complications were assessed for the urinary diversion procedure. Multiple factors, including the type of cancer, the patient’s clinical condition, the severity of the urinary obstruction, surgeon preferences, and the availability at the time of diversion, determined the elected procedure. Antibiotic prophylaxis for each procedure was selected based on local hospital protocols, mostly including quinolones and cephalosporins.

We reported whether the patients received any oncological treatment and their intent (curative or palliative). Follow-up was calculated from the date of cancer diagnosis until the last visit to the office. Renal function was evaluated based on serum creatinine levels at three different moments: before urinary diversion (T0), up to 30 days after diversion (T1), and the most recent level (T2). The patients were stratified regarding their initial creatinine levels into two groups, less and over 4 mg/dl, and its improvement after diversion was stratified in percentiles for a better comparison. Mortality after diversion was also measured.

We used mean and median values and standard deviation for the descriptive statistical analysis of the numerical variables. Categoric variables were demonstrated as frequency (n), percentage (over the entire sample), and valid percentage (excluding those missing). The inferential analysis was accessed using the X2 test, the T-test for independent variables, the ANOVA method, the post hoc test, Pearson’s correlation coefficient, and the Kruskal–Wallis non-parametric test. According to the Kaplan–Meier method, survival curves were demonstrated for OS analysis of overall survival. All analyses were performed using the Statistical Package for the Social Sciences (SPSS) version 21.0.

## 3. Results

We selected 420 patients with a median follow-up of 20.4 months, 250 women, and 170 men (59.5% and 40.5%, respectively), and a mean age of 58.70 years, 18–90 years. All the patients underwent urinary diversion between January 2013 and July 2022. The data on patient demographics and the aspects of their urinary obstruction, type of diversion procedure, and complications are described in Table 1.

Most cases of lower urinary tract (LUT) obstruction were caused by non-urological neoplasms (284 patients, 67.6%); the remaining 136 patients (32.4%) had urological neoplasms. The distribution of the primary neoplastic sites is described in Table 2. The main reason for the suspected diagnosis was acute renal failure, which was observed in 35.7% of the cases (150 patients). Tumor recurrence (40 patients) and pain (31 patients) were the other findings associated with the condition, corresponding to 9.5% and 7.3% of the patients, respectively. However, most patients (180, 42.9%) did not present with any symptoms, and UUT obstruction was an incidental finding during follow-up.

We measured serum creatinine as a parameter of renal function at three different time points: before urinary diversion (T0), up to 30 days after diversion (T1), and the most recent time point (T2); the mean values of all groups were 3.45 mg/dl, 1.84 mg/dl, and 2.59 mg/dl, respectively. In univariate analysis, we did not observe a statistical correlation between age and pre- and post-drainage creatinine values. However, there was a correlation between age and creatinine level at T2 (*p* = 0.03). We found that the mean creatinine level in men was significantly higher in all three groups, with values of 3.82, 2.0, and 2.92 mg/dl, respectively, versus 3.19, 1.72, and 2.28 mg/dl among the women (*p* = 0.05, *p* = 0.05, and *p* = 0.01, respectively). Table 3 summarizes all of the data regarding renal function.

In our sample, 300 patients received some form of antineoplastic treatment, corresponding to 71.4% of the treated group. Of these, 195 patients received palliative treatment, and 105 received treatment with curative intent. The remaining 120 patients (28.6%) did not receive additional cancer-related treatments and were only maintained with comforting and supportive measures owing to their clinical condition (untreated group). There was no significant difference between the groups regarding age, obstruction laterality, dilation degree, and tumor histology (urological or non-urological), with *p*-values of 0.88, 0.40, 0.67, and 0.67, respectively. There was a significant difference between sexes, and the indication for treatment in males was higher than in females (76.5% vs. 68% of the patients, respectively; OR = 1.53; 95% CI = 1.00 − 2.38, X2 test). Surgery and radiotherapy (combined with or without chemotherapy) were more frequently indicated in patients treated with curative intent (*p* < 0.001). Other systemic treatments, including chemotherapy, immunotherapy, and hormone therapy, were administered more frequently in the palliative treatment group (*p* < 0.001).

Data on overall mortality after urinary diversion was obtained from 414 patients; of these, 265 patients died (64%), with a mean time of 251.87 days after the procedure (SD = 313.28 days). The univariate analysis showed no significant correlation between age, sex, and death (*p* = 0.20 and *p* = 0.34, respectively).

We analyzed the mortality of patients with urological and non-urological malignancies and found that 74 of 134 patients in the first group (55.2%) and 191 of 280 patients in the second group died (68.2%). According to the X2 test, patients in the group with non-urological malignancies had a 74% greater probability of mortality than those with urological malignancies (OR = 1.74; 95% CI = 1.14–2.66; *p* < 0.001). Among urological malignancies, patients with prostate cancer had a significantly longer mean overall survival after the diversion procedure than patients with bladder cancer (*p* = 0.05). This difference was not significant among patients with non-urological neoplasia.

When we evaluated only the patients who died, comparing the mean time between urinary diversion and death, we obtained a mean survival rate of 237.05 and 257.68 days, respectively, for patients with urological and non-urological neoplasms. Still, the difference was not significant (*p* = 0.67). Mortality after the diversion was also evaluated for the most prevalent histological subtypes, summarized in Table 4. When assessing the four primary sites with the highest frequency of UUT obstruction (cervix, bladder, colorectal, and prostate), which together corresponded to 83.6% of the total number of patients, we found the most significant survival rate among patients with prostate cancer (367.4 days), and the lowest rate among patients with bladder cancer (158.6 days).

We also evaluated mortality between types of treatment, that is, curative or palliative. Of the 105 patients treated with curative intent, only 33 (31.42%) died, with a mean time between intervention and death of 315.82 days. In the palliative treatment group, 137 of 195 patients (70.25%) died, with a mean time of 348.67 days between intervention and death (*p* = 0.67).

**Figure 1:**
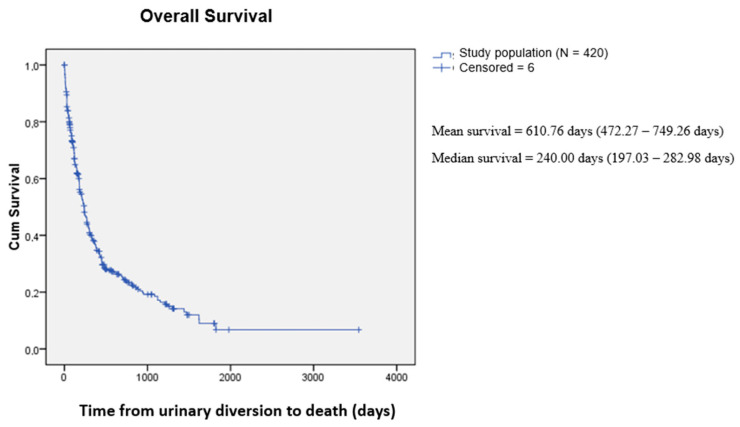
Overall survival.

**Figure 2:**
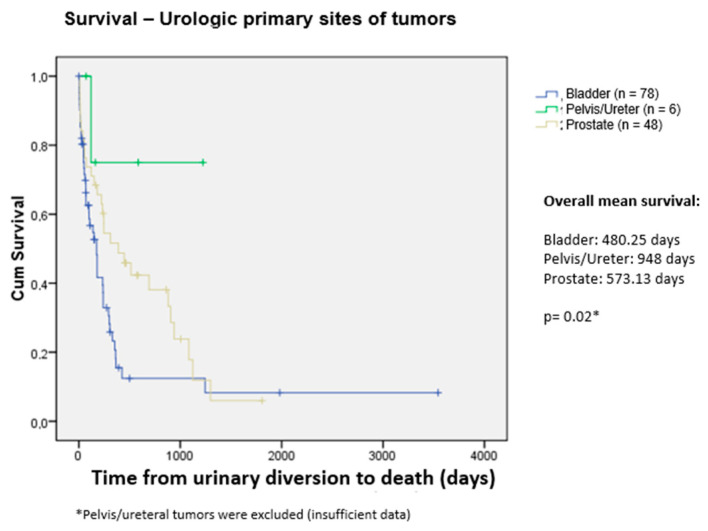
Survival—urologic primary sites of tumors.

**Figure 3:**
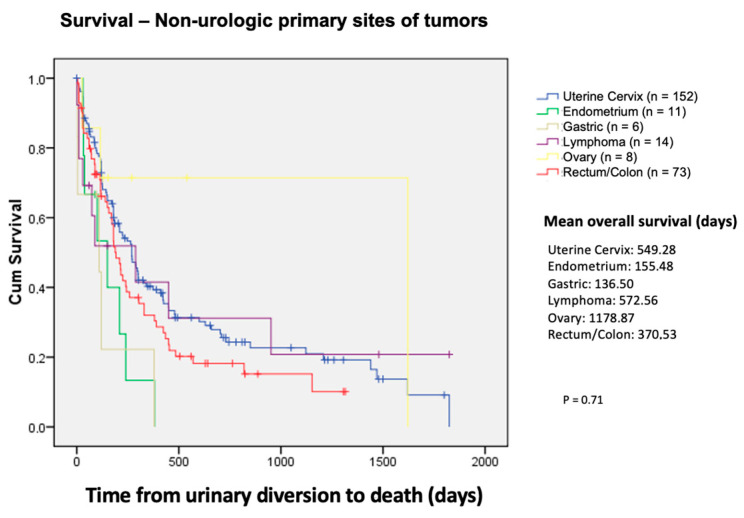
Survival—non-urologic primary sites of tumors.

**Figure 4:**
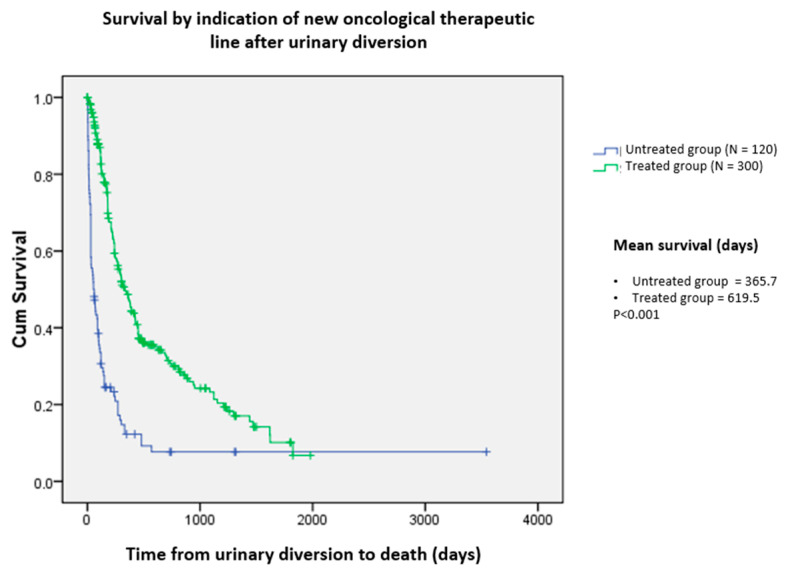
Survival by indication of new oncological therapeutic line after urinary diversion.

The mean overall survival was 610.76 days, according to the Kaplan–Meier method (95% CI = 472.27–749.26). The median was 240.00 days (95% CI = 197.03–282.98)—Figure 1. We also compared survival between the tumor types (urological and non-urological) and their most prevalent histological subtypes. We found that bladder and prostate cancer had an overall mean survival of 480.25 and 573.13 days, respectively, with *p* < 0.02 (Figure 2). For the non-urological group, ovary, lymphoma, and uterine cervix had the highest survival rates of 1178.87, 572.56, and 549.28 days, respectively. Colon, endometrium, and gastric cancer had the lowest survival rates, corresponding to 370.53, 155.48, and 36.55 days, respectively (Figure 3)The survival of patients in the treated group was significantly higher than that of the untreated group: 619.5 and 365.7 days, respectively; *p* < 0.001) (Figure 4).

**Figure 5:**
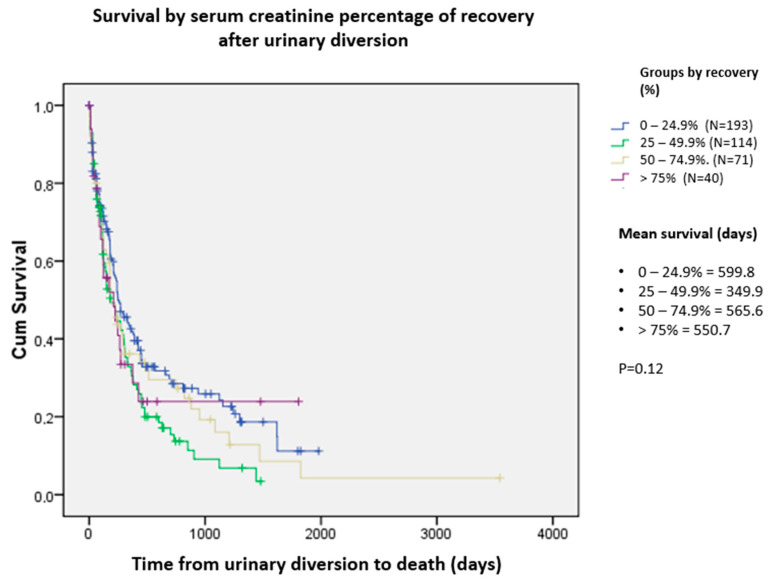
Survival by serum creatinine percentage of recovery after urinary diversion.

We also evaluated the impact of improved creatinine levels on survival, dividing the patients into four groups by the percentage of improvement: 0–24.9%: 25–49%, 50–74.9%, and greater than 75% improvement. No significant differences were observed between the groups (Figure 5).

## 4. Discussion

The objective of our study was to demonstrate that urinary diversion provides a therapeutic opportunity with a consequent gain in survival for most patients with malignant urinary obstruction. In our sample, 300 patients received a specific line of treatment for cancer after urinary diversion, and 25% were treated with curative intent. Only 33 patients died after receiving the new curative therapeutic line. To the best of our knowledge, this is the first study to evaluate the impact of urinary diversion on the indications for new antineoplastic therapeutic lines in patients with malignant ureteral obstruction.

This study showed that 42.9% of patients with hydronephrosis were asymptomatic. In 35.7% of them, acute renal failure was the reason for the diagnosis of ureteral obstruction, and pain was present only in 7.3% of the patients. These findings are surprising since it is an insidious process that favors the adaptation of patients. Lev–Chelouche et al. reported a 37% incidence of asymptomatic hydronephrosis in patients with advanced colorectal cancer [12]. Friedlander et al. reported that ureteral compression due to advanced prostate cancer is an insidious, silent, and sometimes asymptomatic condition that slowly progresses to renal failure [13]. Our study agrees with the literature and thus supports this theory.

Most studies observed significant improvements in creatinine parameters after urinary diversion. Ganatra et al. demonstrated a 57% decrease in serum creatinine levels after urinary diversion [14]. Ishioka et al. reported pre- and post-percutaneous nephrostomy creatinine levels of 4.33 and 1.39 mg/dl, respectively [10]. In a review published by Prentice et al., the mean serum creatinine level improved by >66% [15]. Another study analyzed renal function from the time of drainage to three years later to assess the impact of the presence of a catheter on renal function over time. Although there was a significant drop of up to 50% in the mean creatinine value in the first six months, the study concluded that from then on, there was progressive deterioration in renal function, with creatinine levels increasing to values close to those at the time of drainage, and 57% of patients demonstrating stages 4 and 5 chronic kidney disease after three years [3]. Our sample’s mean creatinine value improved by 46% after drainage. However, according to the literature, the latest mean value showed a more than 40% deterioration, suggesting a progressive worsening of renal function. The causes may be related to disease progression, treatment toxicity, inadequate drainage, and late catheter-related complications.

Our study reported an overall mortality rate of 63.1% in a mean time of 251.87 days after urinary diversion. In a retrospective analysis of 183 patients, Alawneh et al. reported an overall mortality rate of >86% and a median survival of 5 months [16]. Azuma et al. reported a mortality rate of 86.5% at a mean time of 6.4 months in another study of 214 patients [17]. In the present study, patients with ovarian and prostate cancer had the highest survival rates after urinary diversion for over one year. Patients with bladder and stomach cancer had the lowest survival rates, ranging from 3 to 5 months.

In our study, the mean overall survival was 610.76 days. Although patients with urological malignancies had a median survival of approximately seven months longer than those with non-urological malignancies, this difference was not significant. We found a wide range of overall survival rates in the literature, from 1 to 4200 days, with a mean of 190 days (6.4 months). This difference may be due to the diversity of oncological etiologies with different prognoses [10,15,16,17,18].

Our study showed a median survival of 573.13 days (19 months) for patients with prostate cancer, which was significantly longer than that for patients with other urological etiologies. A systematic review of patients with prostate cancer reported survival rates ranging from 2 to 21 months, with a mean of 9 months [19]. In a study of 37 patients with prostate cancer, Chiou et al. reported survival rates of 57%, 29%, and 14% at 1, 2, and 3 years, respectively, with a mean survival of 21 months [20]. Oefelein et al. observed a mean overall survival of 9.2 months among 260 patients, with hormone-naïve patients surviving for 24 months, while patients receiving hormonal therapy survived for seven months [21]. Lapitan et al., in one of the most extensive prospective studies on obstructive uropathy among cervical cancer patients, reported a median survival of 21 weeks (4.9 months) [22]. Dienstmann et al. reported a survival of 8.9 weeks in 50 patients [23]. Noegroho et al. reported a median survival of 5 months in 163 patients [24]. In the present study, patients with ovarian and uterine cervical cancer had significantly higher mean survival rates than patients with other non-urological cancers, 1178 and 549.3 days (39 and 18 months), respectively. These data show survival rates well above the average of those described in the current literature.

Several studies have investigated possible factors related to reduced survival to create a predictive model to support the multidisciplinary team’s decision-making process regarding identifying indications for urinary diversion in patients with malignant obstructive uropathy. Factors such as low albumin levels, severe hydronephrosis, a high number of malignancy-related events, and the presence of metastases and ascites have been associated with worse survival [10,16,17,18,22]. In a study of 50 patients with advanced cervical cancer, Dienstmann et al. analyzed the impact of creatinine levels on overall survival and found no significant correlation [23].

Our study evaluated criteria such as age, sex, histology, creatinine levels after diversion, whether new antineoplastic therapy was indicated, and the presence of metastatic disease at diagnosis. Patients with prostate, ovarian, and cervical cancer who did not present with metastatic disease at diagnosis and who received a new therapeutic line had significantly higher survival rates. When submitted to a new antineoplastic therapeutic line, these patients had almost twice the survival rate of the untreated group. Although one of our hypotheses was that improved renal function is directly related to more prolonged survival, this finding was not confirmed in the present study.

The main limitation of this study is its retrospective nature. However, we know that the context of the clinical conditions of terminally ill patients encompasses ethical and individual issues that hinder the performance of prospective studies of better quality. Once again, the heterogenicity of our sample and the fact that this study was conducted in three different oncologic centers, with different diagnostic and therapy protocols, can also be stated as a study limitation. However, in most cases it may not make much difference, since tertiary institutions frequently follow international guidelines regarding decision-making in this scenario. For the same reason, we did not focus our efforts on addressing the survival rates, since those are well established in the current literature.

The objective of this study was not to assess the patients’ quality of life undergoing urinary diversion. We consider this a limitation of this study because the urinary diversion procedure can cause considerable symptoms that are often the reason for frequent hospitalizations. These hospitalizations keep terminal patients away from their homes and families, impairing their quality of life.

## 5. Conclusions

Our study concluded that urinary diversion in patients with malignant ureteral obstruction contributes to implementing new oncological therapies in a high percentage of cases, helping to provide a cure and survival improvement. Factors such as histological type, type of treatment instituted, and presence of metastatic disease at diagnosis should be considered when deciding the indication for urinary diversion.

## Figures and Tables

**Table 1:** Data on the demographics, urinary diversion procedures, and complications of the study population.

Variable	Subcategories	N	%
Sex	Female	250	59.52
	Male	170	40.48
Ethnicity	Caucasian	241	57.38
	Black	84	20.00
	Brown	41	9.76
	Not reported	54	12.86
Comorbidities	Arterial Hypertension	136	32.38
	Diabetes	60	14.28
	Dyslipidemia	8	1.90
	Obesity	6	1.42
	Smoking	86	20.47
	Others	84	20.00
	Not reported	40	9.55
Laterality	Right side	118	28.1
Of Obstruction	Left side	114	27.1
	Bilateral	188	44.8
Kidney Units	Right side	306	50.3
Compromised	Left side	302	49.7
	Total	608	100.0
Urinary diversion	Ureteral stent	423	69.6
Procedure by	Nephrostomy	176	28.9
Kidney Unit	Not drained	9	1.5
Complications	Infection	25	71.4
	Bleeding	5	14
	Urinary tract perforation	3	8.3
	Nephrectomy	1	2.9
	Vaginal fistula	1	2.9
	Total	35	100.0

**Table 2:** Primary neoplastic sites.

Type of Neoplasm	Etiology	No.	% of Total	Relative %
*Urological*	Bladder	78	18.6	57.4
	Prostate	48	11.4	35.3
	Pelvis/Ureter	6	1.4	4.4
	Testicle	2	0.5	1.5
	Kidney	1	0.2	0.7
	Urethra	1	0.2	0.7
	**Total** 1	136	32.4	100
*Non-urological*	Uterine cervix	152	36.2	53.9
	Rectum/Colon	73	17.4	25.9
	Lymphoma	14	3.3	5.0
	Uterus/Endometrium	11	2.6	3.9
	Ovary	8	1.9	2.8
	Stomach	6	1.4	2.1
	Mama	5	1.2	1.8
	Retroperitoneum	2	0.5	0.7
	Sarcoma	2	0.5	0.7
	Liposarcoma	2	0.5	0.7
	Peritoneum	2	0.5	0.7
	Neuroendocrine	2	0.5	0.7
	Pancreas	2	0.5	0.7
	Schwanoma	2	0.5	0.7
	Vagina	1	0.2	0.4
	**Total**	284	67.6	100

**Table 3:** Data referring to serum creatinine values (mg/dl) in the different groups evaluated.

	Variable	Pre-Drainage Creatinine (T0)	Post-Drainage Creatinine (T1)	Latest Creatinine (T2)
Sex	Male Female *p* value	3.8240 3.1964 0.05	2.0085 1.7266 0.05	2.9204 2.3805 0.01
Type of neoplasm	Urological	4.2598	2.0178	2.8374
	Non-urological	3.0629	1.7560	2.4849
	*p* value	0.001	0.08	0.12
Primary site	Bladder	4.5531	2.0051	2.9508
	Prostate	4.0006	2.1951	2.8568
	Cervix	3.5407	1.8625	2.4044
	Colorectal	2.1874	1.4985	3.0089
Stage of the disease	Metastatic	3.3631	1.8799	2.5827
	Locally advanced	3.4887	1.8236	2.6062
	*p* value	0.71	0.71	0.91
Obstruction Laterality	Bilateral	4.8325	2.3992	3.1101
	Unilateral	2.3299	1.3813	2.1789
	*p* value	<0.001	<0.001	<0.001
Type of procedure	Percutaneous nephrostomy	5.1368	2.2253	3.1743
	Double J	3.4109	1.8687	2.4869
	*p* value	<0.001	0.07	0.01
Patient Current	Alive	3.2226	1.6172	1.6567
Status	Dead	3.5536	1.9565	3.1382
	*p* value	0.32	0.01	<0.001

**Table 4:** Mortality after urinary diversion (days) in patients stratified by histological type.

Urological Neoplasms	N	Average (Days)	Standard Deviation	Non-Urological Neoplasms	N	Average (Days)	Standard Deviation
Bladder	45	158.58	205.750	Uterine cervix	96	292.00	356.236
Prostate	28	367.36	400.111	Rectum/colon	54	209.09	212.315
Pelvis/ureter	1	120	-	Lymphoma	9	211.56	316.908
*Total*	*74*	237.05	308.583	Uterus/endometrium	8	147.88	124.246
				Stomach	5	123.20	153.521
				Ovary	3	588.00	896.600
				Total	175	255.94	320.129

## Data Availability

The data presented in this study are available in this article.
